# Supplementary figures and images for: Establishment of an Indirect ELISA Method for the Detection of the Bovine Rotavirus VP6 Protein
Source: Animals (Basel). 2024 Jan 15;14(2):271. doi: 10.3390/ani14020271 (PMC10812791; doi:10.3390/ani14020271)

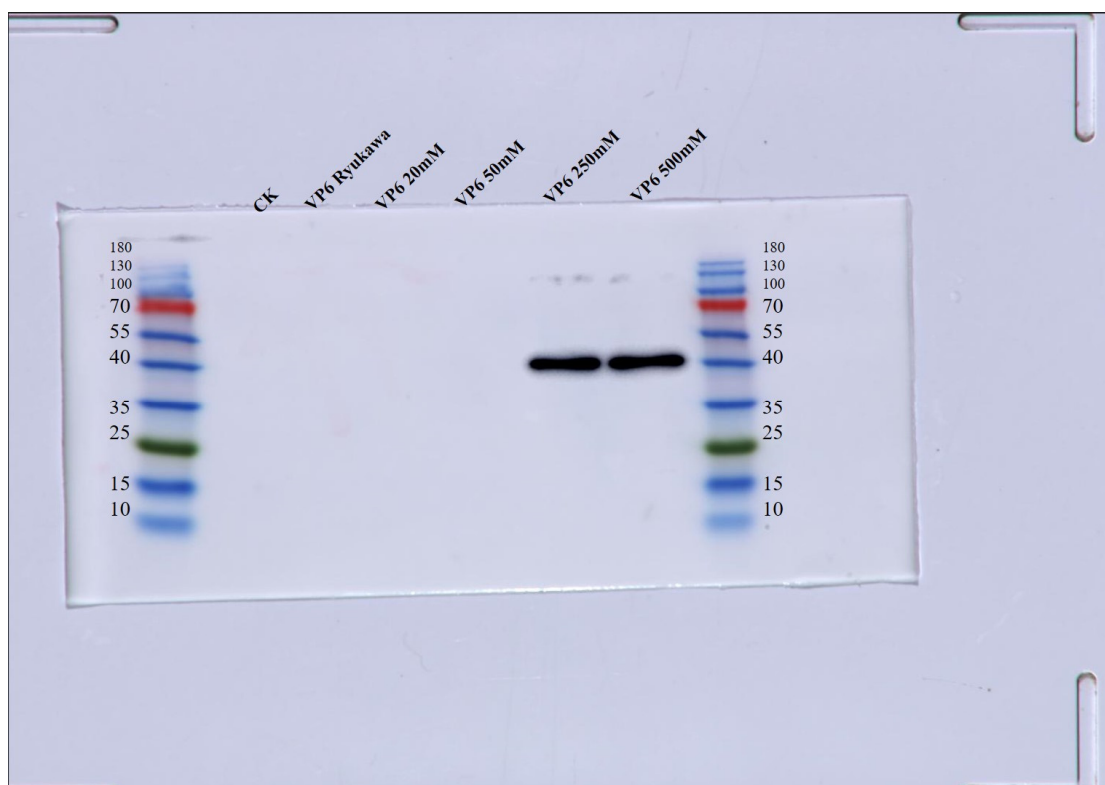

Supplement: Supplementary file 1 [file animals-14-00271-s001.zip › animals-2736687-supplementary.pdf]
